# Electrochemical Analysis of Amyloid Plaques and ApoE4 with Chitosan-Coated Gold Nanostars for Alzheimer’s Detection

**DOI:** 10.3390/bios14100510

**Published:** 2024-10-17

**Authors:** Min-Kyung Shin, Ariadna Schuck, Minhee Kang, Yong-Sang Kim

**Affiliations:** 1 Department of Electrical and Computer Engineering, Sungkyunkwan University, Suwon 16419, Republic of Korea; zkzkzk189@g.skku.edu; 2 Biomedical Engineering Research Center, Smart Healthcare Research Institute, Samsung Medical Center, School of Medicine, Sungkyunkwan University, Seoul 06351, Republic of Korea; minhee.kang@samsung.com; 3 Department of Medical Device Management and Research, Samsung Advanced Institute for Health Sciences & Technology (SAIHST), Sungkyunkwan University, Seoul 06351, Republic of Korea

**Keywords:** Alzheimer’s disease, chitosan, gold nanostars, amyloid-β, apolipoprotein E4, electrochemical detection

## Abstract

Monitoring the progression of Alzheimer’s disease (AD) is crucial for mitigating dementia symptoms, alleviating pain, and improving mobility. Traditionally, AD biomarkers like amyloid plaques are predominantly identified in cerebrospinal fluid (CSF) due to their concentrated presence. However, detecting these markers in blood is hindered by the blood–brain barrier (BBB), resulting in lower concentrations. To address this challenge and identify pertinent AD biomarkers—specifically amyloid plaques and apolipoprotein E4 (ApoE4)—in blood plasma, we propose an innovative approach. This involves enhancing a screen-printed carbon electrode (SPCE) with an immobilization matrix comprising gold nanostars (AuNSs) coated with chitosan. Morphological and electrical analyses confirmed superior dispersion and conductivity with 0.5% chitosan, supported by UV–Vis spectroscopy, cyclic voltammetry, and Nyquist plots. Subsequent clinical assays measured electrical responses to quantify amyloid-β 42 (Aβ42) (15.63–1000 pg/mL) and APoE4 levels (0.41 to 40 ng/mL) in human blood plasma samples. Differential pulse voltammetry (DPV) responses exhibited peak currents proportional to biomarker concentrations, demonstrating high linear correlations (0.985 for Aβ42 and 0.919 for APoE4) with minimal error bars. Cross-reactivity tests with mixed solutions of amyloid-β 40 (Aβ40), Aβ42, and ApoE4 indicated minimal interference between biomarkers (<3% variation), further confirming the high specificity of the developed sensor. Validation studies demonstrated a strong concurrence with the gold-standard enzyme-linked immunosorbent assay (ELISA), while interference tests indicated a minimal variation in peak currents. This improved device presents promising potential as a point-of-care system, offering a less invasive, cost-effective, and simplified approach to detecting and tracking the progression of AD. The substantial surface binding area further supports the efficacy of our method, offering a promising avenue for advancing AD diagnostics.

## 1. Introduction

Alzheimer’s disease, a neurodegenerative disorder, is marked by the buildup of amyloid plaques and neurofibrillary tangles impacting the central nervous system [1,2,3]. The irreversible dementia symptoms caused by AD are noticeable among elderly people (>65 years old), and AD is rarely diagnosed in its early stages, instead being diagnosed only when the brain damage is significant enough to affect the patient’s life [4,5]. The high levels of amyloid plaques in the brain lead to amyloidosis, which can cause toxic damage to nerve cells and contribute to several neurodegenerative diseases [1]. Knowing the concentration of these plaques can aid in the early diagnosis of AD, especially when combined with other biomarkers, to delay or reduce the aggravation of symptoms and improve therapeutic strategies [5,6]. When diagnosing AD, cerebrospinal fluid (CSF) is used for the detection of amyloid plaques because of the high concentration of amyloid-β (Aβ) peptides in the liquid around the brain and the spinal cord [5,7]. Two forms of Aβ, Aβ40 and Aβ42, have been extensively studied due to their association with Alzheimer’s disease [1,8]. The difference between Aβ40 and Aβ42 lies in the length of the peptide; Aβ40 is 40 amino acids long, while Aβ42 is 42 amino acids long [9]. Although both forms of Aβ are present in the brains of individuals with Alzheimer’s disease, Aβ42 has been shown to be more toxic and to form more aggregates, or clumps, in the brain [5,10]. This accumulation of Aβ42 is believed to play a critical role in the development and progression of Alzheimer’s disease [3,11,12]. The Aβ42 oligomers are still the most attractive biomarker to detect and facilitate clinical trials of disease-modifying therapies for Alzheimer’s disease [6,13].

In addition to amyloid plaques, another biomarker that has been studied for its potential role in AD diagnosis is the ApoE4 isoform of apolipoprotein [14,15]. However, the findings regarding the effectiveness of this protein are inconsistent and require further analysis [3,4,14,16,17,18]. ApoE4 enhances the cytotoxicity of Aβ oligomers, which contributes to the disruption of the blood–brain barrier that separates circulating blood from the brain’s extracellular fluid, thereby impairing the brain’s ability to clear Aβ aggregations [10,12,14,19,20]. Given the influence of ApoE4 on Aβ peptides, this protein significantly contributes to the pathogenesis of Alzheimer’s disease. While prevailing approaches in the literature for detecting Aβ and ApoE4 rely on optical or electrical methods, these techniques often lack the required sensitivity for accurately quantifying these peptides in human blood samples, being mostly effective in CSF samples [4,6,14,17,21,22,23,24]. Another challenge emerges when working with clinical samples, as certain studied devices have only been assessed using synthetic or animal-derived samples [23,24,25,26,27]. This limited scope raises uncertainty regarding the ability of the detection method to accurately quantify the targets at low concentrations in human plasma, given the potential presence of interfering substances that could lead to false-positive results.

Different electrical techniques have been used to increase the accuracy of the detection of Aβ and other targets by modifying the sensing devices, e.g., amperometry, cyclic voltammetry (CV), and square wave voltammetry [4,11,22,23]. However, only a few studies have focused on electrochemical methods for blood-based AD diagnosis while applying different nanostructure materials, e.g., metal quantum dots and nanopillars, to enhance the performance of the sensing systems [4,22,24,28,29]. The nanostructures ensure augmented sensitivity due to their high surface area, primarily serving as labels for the AD biomarkers [17,22,23]. In the literature, it is noticeable that most of the studies focus on the detection of the Aβ oligomers, and in some cases target the Tau protein as well [3,6,17,23,30]. However, there is a limited number of studies addressing the detection of ApoE4, particularly in cases where simultaneous detection is needed alongside Aβ oligomers [4,25,31,32,33].

**Figure 1:**
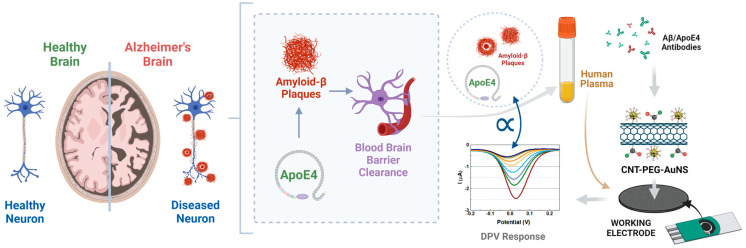
Schematic representation of the proposed methodology for detecting Alzheimer’s disease biomarkers. This study’s overarching concept involves quantifying amyloid plaques and ApoE4 in human blood samples, considering the disruption of the blood–brain barrier (BBB). Plasma is separated from whole blood and analyzed using SPCE devices to quantify low concentrations of Aβ42 and ApoE4. Specific antibodies are conjugated to the CNT-PEG-AuNS-modified working electrodes prior to the assays. A small volume of plasma is applied to the active area of the SPCE, and electrochemical analysis is conducted using the DPV technique, where peak currents correlate with target concentrations.

To address the critical need for early diagnosis of Alzheimer’s disease (AD) and the prevention of severe symptoms, we have enhanced the SPCE devices by incorporating nanostructures to quantify Aβ42 and ApoE4 in blood samples with a high sensitivity, as illustrated in Figure 1. Diagnosing AD based on the detection of Aβ42 in blood plasma is preferable due to its lower invasiveness and increased accessibility [1,6]. Due to the significantly lower levels of Aβ42 in blood compared to CSF, we synthesized an enhanced nanostructure to overcome this limitation and improve the accuracy and sensitivity of Alzheimer’s disease diagnosis [10,22]. To address this limitation and enhance the precision and sensitivity of Alzheimer’s disease detection, we synthesized an enhanced nanostructure, gold nanostars coated with chitosan (AuNS-CHI), to enhance sensitivity and selectivity, while simultaneously promoting stability, improving colloidal dispersion, increasing surface area, and reducing nonspecific interactions. Gold nanoparticles possess excellent physicochemical, thermal, and optical properties, along with a large surface area and biocompatibility, making them highly attractive for modern biomedical applications [34,35]. Chitosan, a biocompatible polymer derived from chitin, further enhances biosensor sensitivity by facilitating the precise immobilization of recognition elements and protecting against interference [36,37]. This immobilization matrix over the working electrodes can further amplify the sensitivity by increasing the surface area available for binding and signal output, thereby improving biosensor performance [38,39]. Our proposed method offers a less invasive and more straightforward means of detecting Aβ42 and ApoE4 in human plasma, enabling the monitoring of Alzheimer’s disease progression, particularly in elderly patients.

## 2. Materials and Methods

### 2.1. Reagents, Materials, and Instrumentation

The materials and methods employed in this study included hydrochloric acid (HCl), silver nitrate (AgNO_3_), chitosan, gold(III) chloride hydrate (HAuCl_4_), phosphate-buffered saline (PBS), potassium ferricyanide (K_3_[Fe(CN)_6_]·3H_2_O), polyvinylpyrrolidone (PVP), and bovine serum albumin (BSA), all sourced from Sigma-Aldrich Corp. (St. Louis, MO, USA). Proteins and antibodies, such as Beta Amyloid Polyclonal Antibody (36-6900), APOE Polyclonal Antibody (PA5-27088), Human Beta Amyloid (1-42) PTD Recombinant Protein (03-111), Human Beta Amyloid (1-40) Recombinant Protein (03-138), Amyloid Beta 42 Human ELISA Kit, Amyloid Beta 40 Human ELISA Kit (KHB3481), and Apolipoprotein E4 Human ELISA Kit (EHAPOE4X10), were obtained from Thermo Fisher Scientific Inc. (Waltham, MA, USA). Electrical measurements were performed using screen-printed carbon electrode (SPCE) devices supplied by QS TAG (Incheon, Republic of Korea) on a CS310 Electrochemical Workstation (Wuhan, China). Microscopy and spectroscopy analyses utilized a Scanning Electron Microscope (SEM) (JEOL JSM-7600F) (Tokyo, Japan) and a Transmission Electron Microscope (TEM) (JEOL JEM 2100F) (Tokyo, Japan), which included energy-dispersive X-ray spectroscopy (EDS). Fourier-transform infrared spectroscopy (FT-IR) was conducted using an Shimadzu IRTracer-100 (Kyoto, Japan) to provide infrared absorption spectra. Data treatment and statistical analysis were carried out using Origin 9.0, and graphical illustrations were created with BioRender.com.

### 2.2. Synthesis of AuNS-CHI Nanostructures

The synthesis of gold nanostars (AuNSs) followed the established protocol from our previous work [40], leading to the formation of unique nanostar-shaped structures. Initially, the AuNSs were synthesized using the seeded-AuNS method described in the literature [34,40]. Briefly, HAuCl_4_ (0.25 mM) was heated in double-distilled water at 95 °C, with 1% trisodium citrate added simultaneously to create the gold seed structures. While stirring, this solution was combined with HAuCl_4_ (0.25 mM) acidified with 1 M HCl, followed by the simultaneous addition of AgNO_3_ (10 mM) and ascorbic acid (100 mM). After the synthesis, the AuNSs were coated with chitosan to form a customized immobilization matrix over the working electrodes. The nanostars were mixed with 0.5% and 1% chitosan solutions in 1% acetic acid and stirred for 90 min at 90 °C. The choice of chitosan for the coating, as opposed to other polymers examined in previous studies, was motivated by our aim to develop a sensor with enhanced sensitivity for detecting low concentrations of amyloid plaques and ApoE4. Chitosan not only provides stability at room temperature but also improves dispersion [37]. After centrifugation at 10,000 rpm for 60 min, excess chitosan was removed, and the particles were resuspended in 1 mL of deionized water.

### 2.3. Enhancement and Characterization of Working Electrodes

After synthesizing the enhanced AuNS-CHI nanostructures, the solution was utilized to modify the working electrodes of the SPCE device via electrodeposition. Following the drop-casting of 60 µL of the AuNS-CHI solution, cyclic voltammetry (CV) was performed over 10 cycles at a scan rate of 150 mV/s, spanning a potential range of −0.7 to 0.7 V. After the measurements, the devices were rinsed with deionized water and dried with nitrogen for electrical analysis. The electrodes were prepared using similar electrodeposition methods, but with different CNTs resulting from various chemical modifications. The electrical characterization was then conducted on electrodes modified with the following CNT solutions: acidified with H_2_SO_4_/HNO_3_ (CNT-COOH), doped with SOCl_2_ (CNT-Cl), functionalized with PEG (CNT-PEG), and decorated with AuNS-PEG (CNT-AuNS-PEG). The initial electrochemical analysis was performed using the CV technique, with a scan rate of 100 mV/s over a potential range of −0.2 to 0.4 V. The buffer solution comprised 5 mM K_3_[Fe(CN)_6_] and 0.1 M KCl as the supporting electrolyte. Also, the CV measurements were performed at different scan rates to measure the response of the device modified with the AuNS-CHI.

### 2.4. Experimental Protocol

The detection of Aβ42 and ApoE4 was conducted using DPV with a scan rate of 100 mV/s over a potential range of −0.1 to 0.3 V in an electrochemical analyzer. Initially, 10 µL of each specific antibody was immobilized on the working electrodes for 15 min at room temperature prior to the electrical assays, after which the electrodes were rinsed with PBS to remove any unbound antibodies. Following this, 1× PBS solutions spiked with varying concentrations of Aβ42 and ApoE4 were measured sequentially using the DPV method. A similar procedure was applied to clinical samples, including CSF and human plasma (healthy subjects) provided by Samsung Medical Center (Institutional Review Board (IRB): IRB-2021-01-036). Each assay was performed using 10 µL of each sample in the modified devices and repeated in at least three devices to ensure reproducibility. Finally, the results obtained with this method were compared to those from conventional Human Amyloid-β 42 and ApoE4 solid-phase sandwich ELISA tests.

## 3. Results and Discussion

### 3.1. Characterization and Analysis of CNT-AuNS-PEG Devices

**Figure 2:**
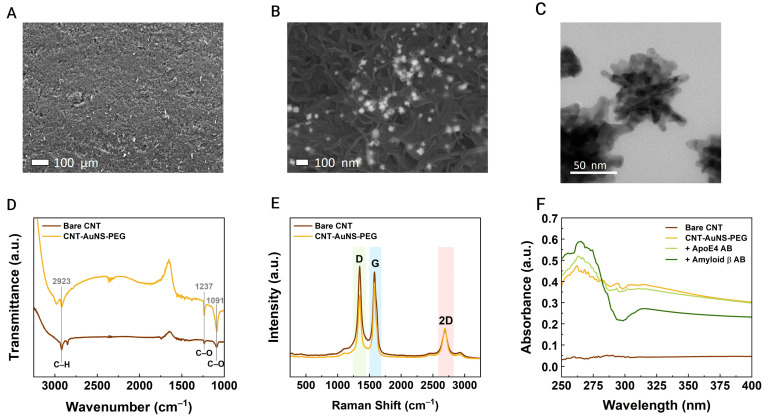
Morphological characterization: Analysis of enhanced carbon nanotubes decorated with PEG-coated gold nanostars. SEM images of CNT-AuNS-PEG at various magnifications: (**A**) ×100, (**B**) ×80 k, and (**C**) ×330 k. (**D**) FTIR spectra and (**E**) Raman spectra comparing bare CNTs with the enhanced structures (CNT-AuNS-PEG). (**F**) UV–Vis absorption spectra of CNT-AuNS-PEG nanocomposites before and after the immobilization of Alzheimer’s disease-specific antibodies.

The SPCE sensors were evaluated after each chemical modification step of the CNTs to confirm enhancements in electrical properties, stability, and dispersion. The electrical characterization included cyclic voltammetry and electrical impedance spectroscopy (EIS) measurements to assess conductivity. Before these electrical tests, the morphology of the nanocomposites was analyzed using various techniques, including SEM, TEM, EDS, UV–Vis spectroscopy, FT-IR, and Raman spectroscopy. The structure and dispersion of the doped CNTs decorated with AuNS-PEG were examined with SEM. As shown in Figure 2A,B, the nanotubes exhibited a uniform shape, with AuNS-PEG dispersed around the carbon material. According to the SEM images in Figure S1, the carbon nanotubes had an average diameter of approximately 17 nm, and there was no significant change in size or shape following each chemical modification. The SEM analysis revealed improved dispersion of the modified nanotubes across the electrode surface, likely due to the presence of PEG; however, further evidence, such as contact angle measurements, would be necessary to confirm any changes in hydrophilicity relative to the original CNTs.

The size of the PEG-coated gold nanostars was approximately 50 nm, as determined from the TEM images shown in Figure 2C. EDS elemental analysis in Figure S2 confirmed the presence of chlorine and gold in the doped CNTs. The low atomic concentration of Cl is likely due to residual chlorine introduced during the CNT doping process with SOCl_2_, which forms acyl chloride groups on the CNTs, resulting in CNT-Cl intermediates. Despite thorough washing, trace amounts of Cl can persist through subsequent modifications, including PEG functionalization and AuNS incorporation. FT-IR and Raman spectra were employed to analyze the CNTs before and after modification. According to the FT-IR spectra presented in Figure 2D, the carbon morphology showed minimal alteration following the acidification and doping processes. The presence of carboxylic acid groups remained unaffected, while doping with thionyl chloride was confirmed by the appearance of peaks, indicating the conversion of carboxylic acid groups to acyl chloride intermediates (CNT-Cl) [41]. This modification increases the reactivity of CNT-Cl, facilitating covalent bonding with antibodies via the acyl chloride groups, which can form stable amide bonds with the primary amine groups present on the antibodies. Moreover, acyl chloride groups may react with other nucleophilic sites, such as hydroxyl or thiol groups, allowing for the attachment of Aβ42 and ApoE4 antibodies specific to Alzheimer’s biomarkers.

The Raman spectra shown in Figure 2E display the characteristic peak bands of the CNTs. The reduction in the intensity of the D band peak before and after CNT modification indicates a decrease in the number of defect states, reflecting a reduction in the amorphous nature of the nanotubes [42]. The UV–vis absorption spectra in Figure 2F show the physical properties of the material where the final nanostructure (CNT-AuNS-PEG) presented a higher absorbance than the bare CNT, indicating a better dispersion. The immobilization of the Aβ42 and ApoE4 antibodies was also evaluated by UV–Vis spectra and the aggregation index (AI) of each antibody was estimated by equation (1) using the absorbance values at the wavelengths of 340 nm ($A_{340}$) and 265 nm ($A_{265}$). The AI values were around 0.7576 and 2.0337 for the Aβ42 and ApoE4 antibodies, respectively. Both indexes were below the upper limit of 10 for both antibodies, indicating a good distribution.

(1) $$AI=\left[\frac{A_{340}}{A_{265}-A_{340}}\right]\times 100$$

**Figure 3:**
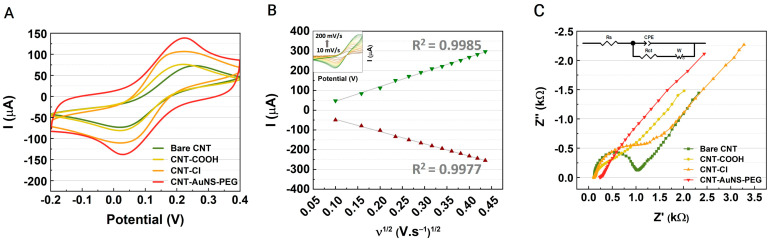
Electrochemical characterization of the SPCE device: Analysis conducted using a buffer solution. (**A**) Cyclic voltammograms (CVs) of bare CNT, CNT-COOH, CNT-Cl, and CNT-AuNS-PEG at a scan rate of 100 mV s^–1^. (**B**) Relationship between cathodic and anodic peak currents and the square root of scan rates (inset: CVs of CNT-AuNS-PEG at various scan rates ranging from 10 to 200 mV s^−1^). (**C**) Nyquist plots for electrodes: bare CNT, CNT-COOH, CNT-Cl, and CNT-AuNS-PEG in 0.1 mol L^−1^ KCl solution containing 5.0 mmol L^−1^ Fe(CN)_6_^3−/4−^ (inset: equivalent circuits with corresponding elements).

To assess the conductivity and stability of the sensing layer with an electrolyte (potassium ferricyanide (5 mM) and potassium chloride (0.1 M)), electrochemical analysis was conducted on the modified SPCE devices using CV. Anodic and cathodic peaks were recorded at each modification step of the working electrode. As illustrated in Figure 3A, the peak current increased following the doping of CNTs with thionyl chloride, while no significant change was observed after PEG treatment. This enhancement is attributed to the improved electrochemical performance of CNTs modified with thionyl chloride compared to CNT-COOH, which is due to a better electron transfer and conductivity. The introduction of acyl chloride groups enhances the dispersion of CNTs and reduces defects that can impede electron flow. In contrast, the carboxyl groups present on CNT-COOH can elevate resistance due to increased oxygen content, which may hinder electron transfer. The highest peak currents were recorded after the final treatment with the CNT-AuNS-PEG nanocomposite, attributed to the superior electrocatalytic effect of the gold nanostructures. The doping effect on the CNTs not only improved conductivity but also enhanced mechanical properties by facilitating denser packing of the nanotubes [43,44]. Also, the CV curves were measured using different scan rates (from 10 to 200 mV/s), and the anodic/cathodic peaks were plotted in Figure 3B against the square root of the scan rate. The currents increased proportionally to the scan rate values with a minimum variation in the peak potential to the right direction, which indicated that the process was diffusion-controlled [45,46].

EIS measurements were conducted to characterize the electrode surface, and the resulting Nyquist plots are presented in Figure 3C. These plots facilitated the evaluation of the sensing system’s impedance across different frequencies. The inset equivalent electrical circuit was utilized to fit the impedance curves. The Randles circuit was selected as the model due to the presence of a diagonal line with an approximate 45° slope, which indicates the Warburg impedance element (W). The circuit model also incorporates ionic resistance (R_s_), double-layer capacitance (CPE), and charge transfer resistance (Rₒ). The charge transfer resistance (R_CT_) of the working electrode with the final nanocomposite (CNT-AuNS-PEG) was measured at 197.5 Ω, significantly lower than the bare electrode at 829.90 Ω, indicating faster electron transfer kinetics.

Interestingly, the R_CT_ observed for CNT-Cl (197.5 Ω) is higher than that of bare CNT, which may seem contradictory, especially considering that the CV peak currents for both materials exhibit similar trends. This discrepancy arises from the fact that while R_CT_ reflects the overall resistance to electron transfer at the electrode interface, CV peak currents are influenced by charge transfer kinetics and the diffusion of species within the electrolyte. Thus, even with a higher R_CT_ for CNT-Cl, enhancements in effective surface area and conductivity due to functionalization can still facilitate adequate current responses during CV measurements. Furthermore, the introduction of acyl chloride groups in CNT-Cl may improve interactions with the electrolyte, contributing to the observed current response.

The doped CNT decorated with AuNS-PEG presented a higher conductivity than the previous modifications of the CNT. Characterization of the nanostructures confirmed that AuNSs-PEG enhances stability and reduces nonspecific interactions of CNTs, while also amplifying the electrical signal to achieve higher sensitivity detection.

### 3.2. Electrochemical Immunoassays for Alzheimer’s Disease Biomarkers

**Figure 4:**
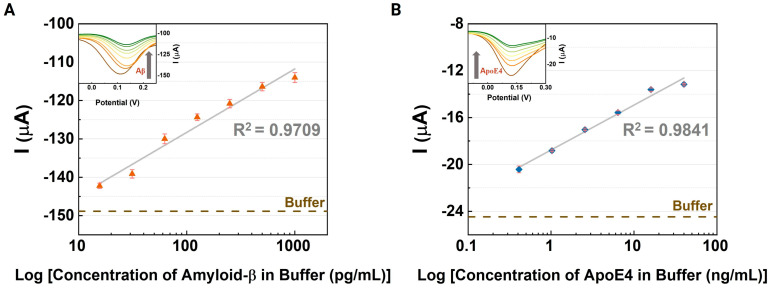
Evaluation of the CNT-AuNS-PEG active layer: Investigation of its effectiveness in detecting Alzheimer’s disease-related biomarkers in a 1× PBS buffer solution by analyzing the variation in peak currents from differential pulse voltammetry (DPV) measurements for (**A**) amyloid-β and (**B**) ApoE4. The inset graphs display results at different concentrations on a logarithmic scale. Error bars indicate the standard deviation from measurements obtained across three devices.

Differential pulse voltammetry was employed to detect various concentrations of Alzheimer’s disease biomarkers in buffer solutions. The assays were conducted with a scan rate of 100 mV/s, using a solution of 5 mM K_3_[Fe(CN)_6_] and 0.1 M KCl as the electrolyte. For each assay, concentration ranges were chosen based on the calibration curves used for ELISA laboratory tests: 15.63 to 100 pg/mL for Aβ42 and 0.41 to 40 ng/mL for ApoE4. Prior to electrical measurements, 10 µL of each AD-specific antibody (100 ng/mL) was immobilized on the sensing devices at room temperature for 1 h. Then, the phosphate-buffered saline (PBS) solutions spiked with the target analytes were introduced over the nanocomposites where the AD-specific antibodies were immobilized and the DPV curves were recorded. At least three devices were measured for a reproducibility assay. As shown in Figure 4, the cathodic peaks increased as the concentration of the amyloid peptides and ApoE4 increased. By investigating the correlation between the peak currents and the levels of AD-related biomarkers, we confirmed the linear responses shown in Figure 4, although the coefficients of determination (R^2^) for both biomarkers were 0.9709 for Aβ42 and 0.9841 for ApoE4. While these values indicate a strong correlation, they also suggest that accuracy and application may be limited due to potential saturation effects at high concentrations on the active surface of the electrode.

The limits of detection (LOD) for the buffer solutions containing the targets were calculated using linear fit equations derived from the peak current trends. For the CNT-AuNS-PEG-based device, the LOD values were estimated to be 1.367 pg/mL for Aβ42 and 0.4679 ng/mL for ApoE4. The assays with the buffer solution proved the potential of the proposed sensing system to be tested with a small volume of human samples. Considering that the levels of amyloid plaques can be lower than the values used in the standard ranges of the ELISA assays, the proposed devices were also tested with low levels of both targets. Figure S3 shows an excellent response for concentrations below the standard ranges, with LOD values estimated to be approximately 0.2042 pg/mL for Aβ42 and 0.853 ng/mL for ApoE4. The assays containing low concentrations of amyloid plaques partially reinforce the goal of this work to detect Aβ42 in blood samples directly without needing to use a high-cost sensing platform or detection method.

### 3.3. Evaluation of the CNT-AuNS-PEG Device Using Human Samples

**Figure 5:**
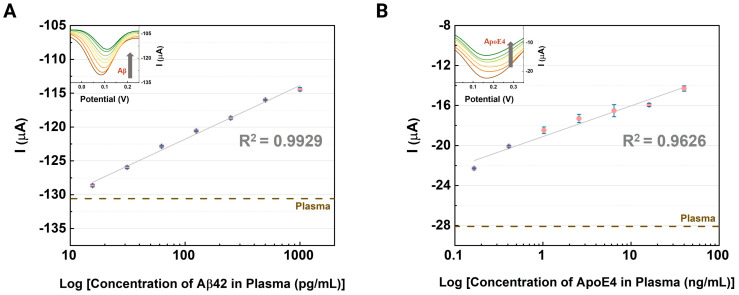
Electrochemical evaluation: Analysis of Alzheimer’s disease biomarkers in clinical samples, illustrating the variation in peak currents with DPV responses. The inset graphs display the responses at different concentrations of (**A**) Aβ42 (15.63–1000 pg/mL) and (**B**) ApoE4 (0.41 to 40 ng/mL) in human plasma, shown on a logarithmic scale. Error bars indicate the standard deviation from measurements taken across three devices.

The same experimental protocol used for buffer assays was applied to measurements with human samples. Prior to DPV measurements, a solution of 3% bovine serum albumin (BSA) in PBS was drop-cast over the active layer following antibody immobilization. The incubation of BSA in the active layer for 15 min helped to reduce nonspecific binding. Next, the surface was cleaned with PBS and dried with N_2_ before electrical measurements of the CSF and the human plasma samples. Subsequently, 10 µL aliquots of human samples containing Aβ42 and ApoE4 were injected onto the sensing area of the devices, and the DPV curves were recorded using the electrochemical analyzer. The peak currents from these DPV measurements for amyloid plaques and ApoE4 biomarkers in plasma samples are shown in Figure 5A and Figure 5B, respectively. All experiments were repeated in triplicate, with average current values plotted along with standard deviation error bars. The inset graphs display the DPV curves for each assay. Both peak currents presented a growth tendency as the levels of the biomarkers increased in the human clinical samples. The coefficients of determination for the Aβ42 and ApoE4 were around 0.9929 and 0.9626, respectively, indicating a linear response for the plasma assays. The human samples had higher values for the current signals because they may originally contain more charged particles from the specific targets. The LOD values for these assays were around 0.2042 pg/mL and 0.6366 ng/mL for the detection of Aβ42 and ApoE4, respectively.

To ensure the effectiveness of the device in detecting amyloid plaques, CSF samples were also utilized as they are the standard sample for AD diagnosis. Comparison of the electrical response for plasma assays in detecting Aβ42 with the results obtained from CSF samples (as shown in Figure S4) revealed that the enhanced nanocomposite exhibited a better sensitivity and accuracy when used with plasma samples. The results of our experiments were also compared with some recently published works that used carbon materials and metal nanoparticles, as shown in Table 1. In comparison to other recently published works that used electrochemical methods for AD diagnosis, our device demonstrated comparable or even a lower limit of detection values for Aβ42 and ApoE4 in the plasma samples. However, what sets our device apart is its low cost and simplicity, as well as the ability to directly use plasma samples without the need for extra chemical steps or dilution. Our device could detect Aβ42 and ApoE4 in plasma samples without extra steps or dilution, while using a simpler and lower-cost sensing system with a rapid response and high sensitivity, suggesting the device has potential as a point-of-care diagnostic tool.

### 3.4. Interference and Cross-Reactivity Assessment

**Figure 6:**
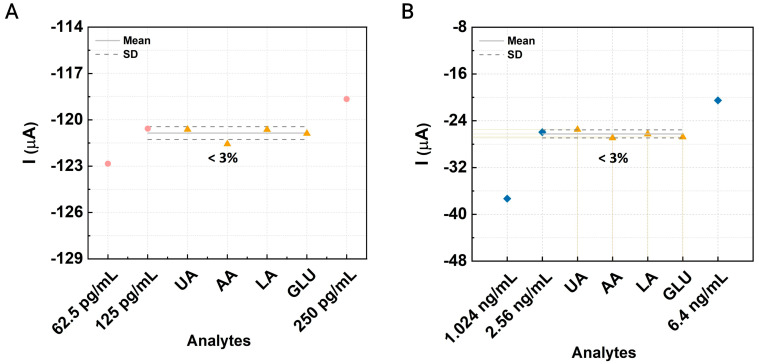
Interference assessment: Evaluation of the impact of various interfering compounds on the CNT-AuNS-PEG device. Differential pulse voltammetry (DPV) peak currents were measured for (**A**) amyloid-β 42 (circle) and (**B**) ApoE4 (rhombus), following the addition of interferents (triangle): 60 µM uric acid (UA), 10 µM ascorbic acid (AA), 50 mM lactic acid (LA), and 0.2 mM glucose (GLU) in plasma samples.

The analytical specificity of the proposed sensing device was assessed through an interference study to determine its susceptibility to false-positive responses caused by potential organic molecules. Plasma samples were used as electrolytes, and interferents were selected based on their high concentrations in human blood. For this test, saturated concentrations of 60 µM uric acid (UA), 10 µM ascorbic acid (AA), 50 mM lactic acid (LA), and 0.2 mM glucose (GLU) were introduced into the plasma sample during DPV measurements. The same electrical parameters were applied, and curves were recorded for specific concentrations of each AD biomarker. Interfering substances were added between the second and third concentrations of the targets. As shown in Figure 6, the peak current response remained proportionally consistent with Aβ42 and ApoE4 levels in human plasma. However, the addition of interferents resulted in only a slight variation (<3%) in the current compared to the previous peak current before the introduction of uric acid. This minimal effect of interfering substances on the electrical signals indicates that the CNT-AuNS-PEG-based device maintains a high analytical specificity for Aβ42 and ApoE4.

**Figure 7:**
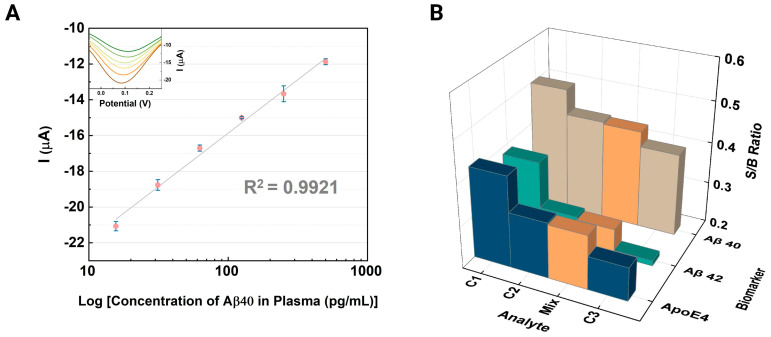
Cross-reactivity assessment: Analysis of the interaction among Alzheimer’s disease biomarkers. (**A**) Differential pulse voltammetry (DPV) peak currents of the CNT-AuNS-PEG when measuring amyloid-β 40 as a potential Alzheimer’s biomarker. The inset graph displays the DPV curves of amyloid-β 40 across different concentrations. (**B**) Evaluation of the enhanced screen-printed carbon electrode (SPCE) for cross-reactivity with samples containing Aβ40, Aβ42, and ApoE4.

We investigated Aβ40 as another promising biomarker for brain amyloidosis using the same sensing platform employed in previous experiments. The peak current response for plasma Aβ40, illustrated in Figure 7A, shows a strong linear correlation, with an R^2^ value of 0.9921. After confirming the device’s ability to target different amyloid alloforms (40 and 42 amino acids long), we conducted a cross-reactivity test with a mixed solution containing ApoE4 (16 ng/mL), Aβ42 (250 pg/mL), and Aβ40 (250 pg/mL). This mixed solution was analyzed alongside varying concentrations of the biomarkers, with C1, C2, and C3 corresponding to 6.4 ng/mL, 16 ng/mL, and 40 ng/mL for ApoE4, and 125, 250, and 500 pg/mL for Aβ42 and Aβ40, respectively. In Figure 7B, the peak currents are presented as signal-to-background (S/B) ratios, calculated from blank measurements to facilitate comparisons among the three biomarkers. The S/B ratios were approximately 1.17%, 2.57%, and 1.03% for ApoE4, Aβ42, and Aβ40, respectively, following the introduction of the mixed solution. The minimal variations in the S/B ratios indicate a low cross-reactivity among the biomarkers, demonstrating the enhanced SPCE device’s high specificity and capability to accurately detect AD biomarkers.

### 3.5. Validation and Recovery Testing

**Figure 8:**
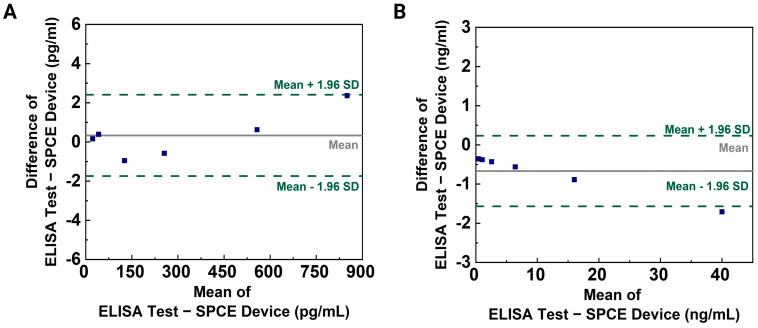
Validation analysis: Comparison between gold-standard ELISA laboratory tests and CNT-AuNS-PEG-based devices. Bland–Altman plots depict the level of agreement for measurements using diluted human plasma samples containing (**A**) Aβ42 and (**B**) ApoE4.

The proposed device was validated through clinical assays using the ELISA test as the gold-standard technique. Human plasma was diluted 1:8 in the standard diluent buffer according to the ELISA protocol, with absorbance measured at 450 nm using a microplate reader. Plasma solutions were prepared using the concentration ranges specified in the ELISA protocol, and aliquots were used for electrical measurements with the modified SPCE devices. Concentrations of Aβ42 and ApoE4 were estimated using a curve-fitting approach for both methods. Bland–Altman plots, shown in Figure 8, demonstrate excellent agreement between the CNT-AuNS-PEG-based device and ELISA tests for low concentrations of Aβ42 and ApoE4 in diluted plasma samples. Variability in the correlation at high analyte concentrations may be attributed to saturation on the working surface of the SPCE devices. However, the concentration range used aligns with the traditional detection method for CSF samples. Since Aβ42 levels in plasma are lower than in CSF, discrepancies at higher concentrations are not significant for these assays. Recovery analysis, detailed in Table 2, presents the detected concentrations for Aβ42 and ApoE4 based on linear equations derived from the plasma assays shown in Figure 5, with recovery values calculated using the following formula:

(2) $$Recovery(\%)=\frac{Detected\;Concentration}{Actual\;Concentration}\times 100$$

This method allows us to assess the accuracy of the sensor by comparing the detected concentrations to the actual known concentrations added to the samples. Although the highest variation was observed for ApoE4, the results from the modified SPCE devices demonstrated a greater accuracy compared to the ELISA recovery tests shown in Table S1, indicating a closer alignment with the actual added concentration values. The high recovery rates observed may be due to the sensor’s enhanced sensitivity, which can sometimes lead to overestimation of the biomarker concentrations, particularly at low levels.

## 4. Conclusions

Alzheimer’s disease is characterized by amyloid plaques and neurofibrillary tangles, with Aβ peptides and ApoE4 being potential biomarkers; our study enhanced an SPCE device’s sensitivity using a CNT-AuNS-PEG nanocomposite for improved detection of these biomarkers in human blood. By investigating electrochemical signals using Aβ42 and ApoE4 in buffer and plasma solutions, we optimized the active layer, demonstrating linear trends between peak currents and biomarker concentrations, even at low levels. The linear tendencies observed in all assays using PBS buffer, CSF, and human plasma demonstrated a high correlation between the peptide concentration and the current peaks from the DPV measurements. The SPCE devices exhibited a high specificity and clinical accuracy, with LOD values of 0.2042 pg/mL for Aβ42 and 0.6366 ng/mL for ApoE4 in undiluted plasma.

Aβ40 was included in cross-reactivity tests to ensure its presence would not interfere with the detection of Aβ42 or ApoE4. The results indicated minimal signal variation (<3%) in the presence of mixed biomarkers, confirming the device’s high specificity. Nonspecific binding of interferents was further minimized through a post-treatment step using BSA. In the presence of relevant interferents, the variation in the current was less than 3%. Also, the agreement test proved that the SPCE device has a high agreement level with the ELISA, as well as better recovery results. This low-cost, disposable sensor holds promise for early AD detection and monitoring, offering a rapid, accurate, and less-invasive alternative to traditional techniques that require large volumes of CSF samples for ELISA assays. Additionally, it has the potential to aid in research studies regarding the relationship between the plasma levels of Aβ42 and ApoE4 and the diagnosis and treatment of AD. Overall, the prospects of this research are significant, and the benefits are far-reaching. The device has the potential to aid in the early detection and diagnosis of AD, as well as in the monitoring of its progression, leading to better patient outcomes.

## Figures and Tables

**Table 1:** Comparison of detection methods: Assessment of various electrical techniques reported in the literature for detecting Aβ42 and ApoE4.

Sensing Material ^a^	Target	Method ^b^	Sample ^c^	Concentration	LOD	Reference
Au Nanopillars	Aβ42	SWV	Artificial Tear	0.1 to 1 ng/mL	0.14 ng/mL	[23]
CdSe@ZnS QDs	ApoE4	SWV	Diluted plasma	10 to 200 ng/mL	12.5 ng/mL	[4]
GO/Au	Aβ42	Transfer Curves	CSF/Plasma	10^−1^ to 10^5^ pg/mL	9990 pg/mL (Plasma)	[24]
CNT	Aβ42	Transfer Curves	Diluted plasma	6.75~4500 pg/mL	9.585 pg/mL	[22]
AuBP@Pt	ApoE4	Amp	Goat Serum	0.05 to 2000 ng/ml	0.015 ng/mL	[25]
AuNPs	Aβ42	LSPR	CSF Buffer	4500~450,000 pg/mL	6750 pg/mL	[31]
ITO/FracAu	ApoE4	Amp	PBS	1.0 to 10,000 ng/mL	0.30 ng/mL	[20]
CNT-AuNS-PEG	Aβ42	DPV	Plasma	15.63 to 500 pg/mL	0.2042 pg/mL	This work.
	ApoE4			0.41 to 40 ng/mL	0.6366 ng/mL	

^a^ Au: gold; CdSe@ZnS: cadmium-selenide/zinc-sulfide; QDs: quantum dots; Pt: platinum; GO: Graphene Oxide; CNT: carbon nanotubes; AuBP: gold nanobipyramid; FracAu: fractal gold; AuNS: gold nanostars; PEG: polyethylene glycol.; ^b^ SWV: square wave voltammetry; Amp: amperometric; LSPR: localized surface plasmon resonance; DPV: differential pulse voltammetry.; ^c^ CSF: cerebrospinal fluid; PBS: phosphate-buffered saline.

**Table 2:** Recovery analysis: Results demonstrating the efficacy of the proposed method using plasma samples.

Biomarker	Added	Detected	Recovery
Amyloid-β 42	31.25 pg/mL	33.74 pg/mL	108%
	125 pg/mL	131.37 pg/mL	105%
	250 pg/mL	236.35 pg/mL	95%
ApoE4	1.024 ng/mL	0.966 ng/mL	94%
	2.56 ng/mL	2.832 ng/mL	111%
	6.40 ng/mL	6.81 ng/mL	106%

## Data Availability

The data that support the findings of this study are available on request from the corresponding authors.

## Supplementary Materials

The following supporting information can be downloaded at https://www.mdpi.com/article/10.3390/bios14100510/s1.
