# Supplementary material for: Electrochemical Analysis of Amyloid Plaques and ApoE4 with Chitosan-Coated Gold Nanostars for Alzheimer’s Detection
Source: Biosensors (Basel). 2024 Oct 17;14(10):510. doi: 10.3390/bios14100510 (PMC11506205; doi:10.3390/bios14100510)
Supplement: Supplementary file 1 [file biosensors-14-00510-s001.zip › biosensors-3222918-supplementary.pdf]

Supplementary Materials

Electrochemical Analysis of Amyloid Plaques and ApoE4 with Chitosan-Coated Gold Nanostars for Alzheimer's Detection

Min-Kyung Shin[a,*], Ariadna Schuck[a,*], Minhee Kang[b,c,**], and Yong-Sang Kim[a,**]

[a]Department of Electrical and Computer Engineering, Sungkyunkwan University, Suwon, Republic of Korea Biomedical Engineering

[b]Biomedical Engineering Research Center, Smart Healthcare Research Institute, Samsung Medical Center, Sungkyunkwan University School of Medicine, Seoul, Republic of Korea

[c]Department of Medical Device Management and Research, SAIHST (Samsung Advanced Institute for Health Sciences & Technology), Sungkyunkwan University, Seoul, Republic of Korea

**Corresponding author: arischuck@skku.edu (A. Schuck); yongsang@skku.edu (Y.-S. Kim). Phone number: +82-31-299-4323. Mailing address: Engineering Building 1 (23), Room 23232, 2066, Seobu-ro, Jangan-gu, Suwon, Gyeonggi-do, Republic of Korea*

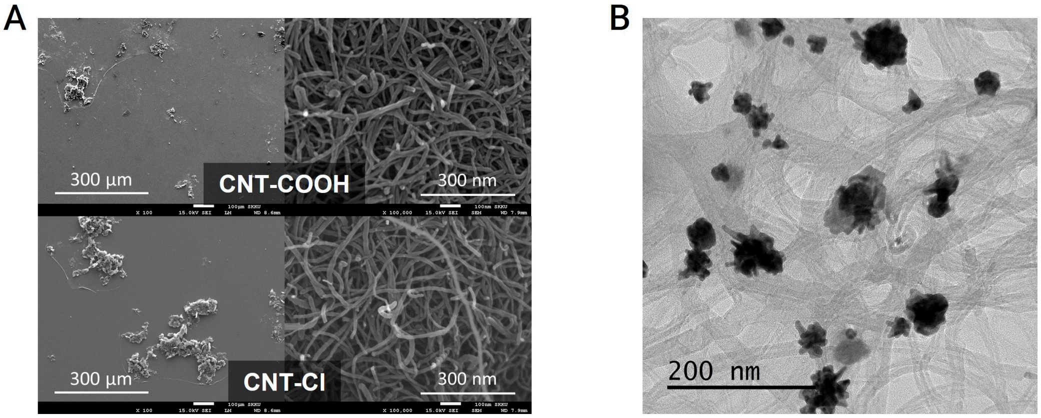

Fig. S1. Microscope images of the surface morphology of the composite electrode between each modification step. (A) SEM images for the functionalization of the CNTs after acidification and doping steps. (B) TEM images of the gold nanostars after the coating process with PEG.

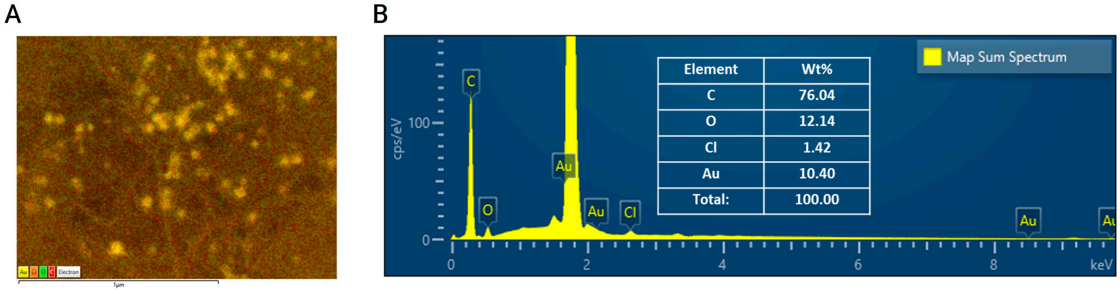

Fig. S2. EDS analysis results for the CNT-AuNS-PEG nanostructure. (A) EDS layered image with the element mapping profiles and (B) map sum spectrum.

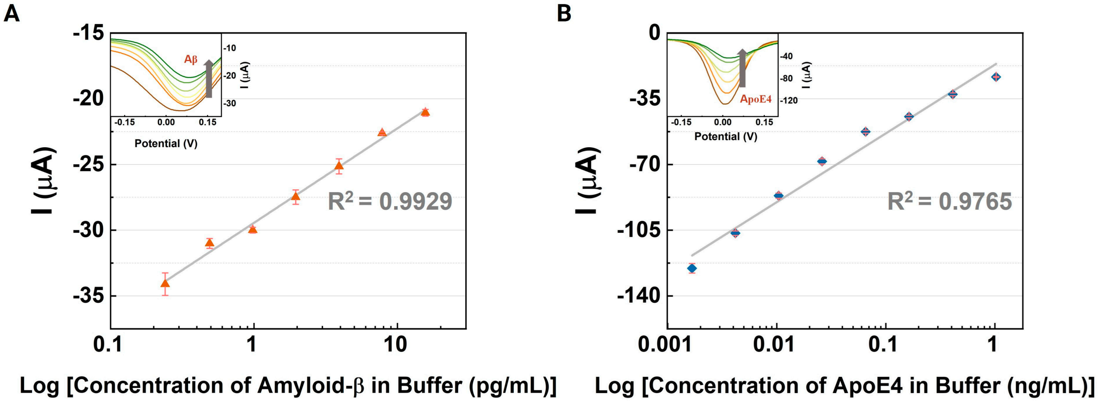

Fig. S3. Evaluation of the CNT-AuNS-PEG devices for detection of low concentrations of the AD-related biomarkers in 1× PBS buffer solution. Variation of the peak currents from the DPV measurements (inset graphs)

for (A) Aβ42 and (B) ApoE4 for various low concentrations on a logarithmic scale. Error bars were calculated as the standard deviation of 3 devices.

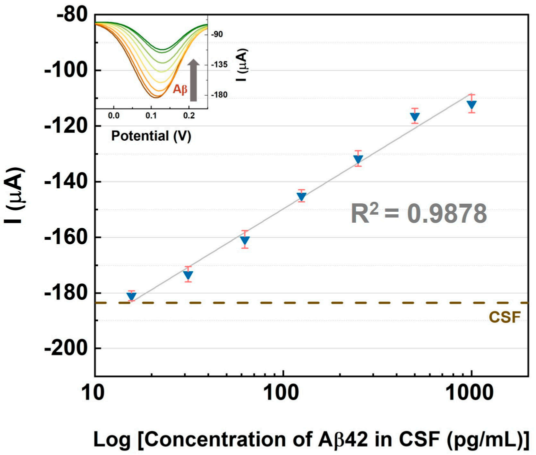

Fig. S4. Electrochemical analysis of Aβ42 in CSF samples. Variation of the peak currents with the DPV responses (inset graphs) for Aβ42 various concentrations (15.63 - 1,000 pg/mL) on a logarithmic scale. Error bars were calculated as the standard deviation of 3 devices.

Table S1. Recovery results of the ELISA assays using plasma samples.

Biomarker	Added	Detected	Recovery
Amyloid-β 42	31.25 pg/mL	37.71 pg/mL	121%
	125 pg/mL	121.04 pg/mL	97%
	250 pg/mL	251.99 pg/mL	101%
ApoE4	1.024 ng/mL	1.402 ng/mL	137%
	2.56 ng/mL	2.990 ng/mL	117%
	6.40 ng/mL	6.961 ng/mL	109%
